# Alternative splicing factor RAB3IP as a novel risk signature to predict the prognosis of colorectal cancer

**DOI:** 10.7150/jca.110271

**Published:** 2025-06-23

**Authors:** Zhengwei Zhou, Fei Gao, Han Lei, Haixuan Wen, Jiaxi Tang, Yulong Peng, Lili Fan, Lu Xu, Guang Shu

**Affiliations:** 1 Department of Pathology, School of Basic Medicine, Central South University, Changsha, Hunan, China.; 2 School of Traditional Chinese Medicine, Jinan University, Guangzhou, China.; 3 Department of Pathology, First Affiliated Hospital of Jinan University, Guangzhou 510630, China.

**Keywords:** alternative splicing, colorectal cancer, splicing factor, prognosis, metastasis.

## Abstract

Emerging evidence suggests that aberrant alternative splicing plays a vital role in the development of tumors. However, the expression of splicing factors (SF) in colorectal cancer and its relationship with prognosis is still unclear. Here, we divided patients into high-risk and low-risk groups through univariate COX analysis and LASSO regression analysis, and selected 13 alternative splicing factors that are highly correlated with prognosis for subsequent analysis. We systematically analyzed the prognostic value of transcription levels of SFs in colorectal cancer (CRC) and found that RAB3A interacting protein (RAB3IP), programmed cell death 4 (PDCD4), golgin B1 (GOLGB1), and neuregulin 4 (NRG4) as the most predictive markers for the prognosis of CRC. After comparing the expression of four splicing factors in cancer tissues with normal tissues as well as OS analysis, it is strongly indicated that only RAB3IP demonstrates a significant positive correlation with favorable prognosis. Accordingly, we established a risk signature of transcription levels of RAB3IP as an independent prognostic marker for CRC. Moreover, by the Gene Set Enrichment Analysis (GSEA), we demonstrated that the RAB3IP was correlated to Cell Cycle, WNT pathway and Spliceosome in cancer. In conclusion, our findings demonstrate that SFs play a critical role in CRC pathogenesis, and identify RAB3IP as a novel prognostic biomarker for CRC.

## Introduction

Alternative splicing (AS) is a process necessary for gene transcription into mature mRNA of different variants, and it is also one of the main reasons for protein diversity. AS is essential for tissue development, differentiation and homeostasis, and is a key cellular pathway in higher eukaryotes 1. Whole-genome studies estimate that 90% to 95% of human genes have varying degrees of alternative splicing 2, 3. Most splicing factors are RNA-binding proteins (RBPs) that, in combination with other elements, promote or inhibit splicing site recognition during tissue development and cell differentiation 4. Dysregulation of alternative splicing has been found to be involved in the development of various cancers, where splicing is largely de-regulated due to mutations or abnormal expression of key splicing regulatory proteins 5. Based on the analysis of the cancer genome atlas (TCGA) data of 32 types of cancers in 8705 patients, the occurrence of alternative splicing events in tumors is more than 30% higher than that of normal tissues 6, mainly affecting physiological activities such as cell cycle control, cytoskeletal organization, migration and cell-cell adhesion, etc. More importantly, cancer-specific splice variants contribute to tumor cell survival and cancer progression and may serve as prognostic biomarkers for predicting patient survival 2, 6-9.

Colorectal cancer kills nearly 700,000 people each year, a high mortality rate that makes it the fourth deadliest cancer in the world (after lung, liver, and stomach cancer) 10-12. Increasing evidence indicates have shown that alternative splicing also plays a pivotal role in CRC initiation and progression, with aberrant AS closely linked to colorectal carcinogenesis 11, 13-16. Among them, SRSF6 can directly bind to the exon 23 motif of *ZO-1* to perform abnormal splicing and promote the proliferation and metastasis of CRC 17. HNRNPLL, as a newly discovered colorectal cancer metastasis inhibitor, regulate the alternative splicing of *CD44* during the epithelial-mesenchymal transition, thereby inhibiting the metastasis of CRC 13. In addition, alternative splicing induced by PHF5A hyperacetylation can stabilize *KDM3A* mRNA and promote its protein expression, resulting in a poor prognosis for CRC patients 18. Sam68, an RNA-binding protein, affects cell growth and glycolysis by regulating the alternative splicing and expression of PKM2 in CRC 19. Furthermore, epigenetic modifications can achieve tumor control by regulating alternative splicing 20.

Accumulating evidence indicates that dysregulation of alternative splicing (AS) directly facilitates oncogene activation and enhances neoplastic cell proliferation and metastatic potential in colorectal cancer 21, 22. Recent studies have established AS events as independent prognostic biomarkers in CRC, which has prompted our focused investigation into the mechanistic role of splicing factors in CRC pathogenesis 23. Although there is a large amount of evidence that AS has a direct or indirect role in the occurrence and development of CRC, further research is needed to clarify the specific AS event regulation mechanism and macro-prognosis application. In this study, we drew the alternative splicing map in CRC by analyzing RNA-seq data and assessed the risk ratio of AS events with COX analysis. The gene RAB3IP, which has the strongest correlation with CRC, was noticed through Lasso regression analysis. Functional experiments such as proliferation and migration proved that this gene plays a vital role in the occurrence of CRC and can be used for independent prognostic analysis. Meanwhile, our study provides new strategies for discovering novel biomarkers for prognosis prediction and targets for treatment development.

## Materials and Methods

### AS-related gene datasets and clinical date

In this study, RNA-seq data and complete clinical follow-up records (overall survival (OS) ≥ 30 days) for the colorectal cancer (CRC) cohort were obtained from the TCGA SpliceSeq database (https://bioinformatics.mdanderson.org/TCGASpliceSeq/). CRC samples with incomplete clinical or molecular profiles in the TCGA dataset were stringently excluded, yielding a final analytical cohort of 447 rigorously curated CRC cases. Eligible patients included in this article are in accordance with the following inclusion criteria: (1) pathological confirmation of the diagnosis; (2) no prior history of neoadjuvant therapy or other malignancies; (3) complete clinicopathological data. The detailed clinic parameters of enrolled patients were presented in Table. Exclusion criteria included the following: (1) other treatments were used after the operation; (2) patients with incomplete survival data; (3) other organ tumors. The SpliceSeq tool was used to analyze the AS profile and evaluate the splicing pattern of mRNA in CRC patients. According to the PSI value of alternative splicing (Percent-spliced-in) (0-1), delete unnecessary data, and finally quantify seven AS events: exon skip (ES), mutual Exclusion exon (ME), reserved intron (RI), alternate promoter (AP), alternate terminator (AT), alternate donor site (AD) and alternate acceptor site (AA). Finally, the R language (version 3.4.0) is used for statistical analysis, and the UpSet diagram is used to visualize the various combinations of the intersection of the seven AS types, and clearly show the quantitative results of multiple AS event interaction sets.

### Selection of key genes in AS events and analysis of differential expression

In the AS event, genes with Z-score>2() were identified as candidate genes, and the volcano map was drawn. At the same time, the Lasso regression curve was used to prevent the model from overfitting (λ< -3.3). Perform univariate Cox regression analysis on the expression in the TCGA data set to evaluate the prognostic value of variable splicing candidate genes, select 13 candidate genes that are actually related to survival (P <0.1), and conduct the next functional study. Then, four candidate genes and their coefficients are determined by the minimum standard, and the best penalty parameter λ related to the TGGA data set is selected. Use the following formula to calculate the risk score of the signature 24:

Risk score =
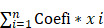


Where Coefi is the coefficient, and x_i_ is the relative expression value of each selected regulator after z-score conversion. This formula is used to calculate the risk score of each patient in the TGGA data set. In CRC cases, high-risk subtypes (samples with a risk score higher than 5) and low-risk subtypes (samples with a risk score lower than 5) are defined based on the risk score of their tumor samples. Based on the risk score, ROC analysis is used to test whether survival predictions are sensitive and specific.

### Subsistence analysis

This study included 447 patients with overall survival (CRC) of at least 30 days. According to the median of each parameter, the patients were divided into two groups, and the AS event data and survival data were combined for single-factor COX analysis. In addition, the Kaplan-Meier curve is drawn by comparing the OS data of CRC patients within 5 years. The Chi-square test is used to compare the difference in survival status between high-risk and low-risk. Then, use receiver operating characteristics (ROC) analysis to compare the efficiency of the survival ROC software package in R for each predictive model.

### Independent prognostic analysis and survival analysis of NRG4, GOLGB1, PDCD4 and RAB3IP

Univariate and multivariate Cox regression analyses were used to obtain the prognostic value of the risk score generated by the multivariate model (P<0.05). Demographic and clinical information (including age, grade, number of nodules, etc.) are used for model calibration. Biomarker Exploration of Solid Tumors (BEST) portal (https://rookieutopia.com/app_direct/BEST/) was used for validation the association between the expression of RAB3IP, PDCD4, GOLGB1 and NRG4 and tumor progression. The mRNA expression level and survival prognosis of the four genes in the cancer group and the normal group in colorectal cancer were analyzed through the UALCAN website(http://ualcan.path.uab.edu/index.html). COPTAP is used to study the differences in protein function of candidate genes, a website dedicated to the study of protein expression levels.

### Gene set enrichment analysis (GSEA) of RAB3IP

The GSEA 3.0 software with genome c2 (cp.kegg.v.6.2.symbols.gmt) was used to perform the prognostic-related gene set enrichment analysis (GSEA) of MeDEG. The mRNA expression level of 447 colorectal cancer patients in the TCGA database is used as a dataset. The number and type of arrangement are set to "1000" and "phenotype" respectively. Enrichment scores> 0.4 and P <0.05 were considered statistically significant.

### Cell culture and transfection

The normal human colon mucosal epithelial cell line NCM460 and human colorectal cell lines HCT116 and SW480 used in this study were purchased from the American Type Culture Collection (ATCC;). The colorectal cell lines HCT8, Caco2, and HT29 were kindly provided by Professor Wancai Yang (Key Laboratory of Precision Oncology of Shandong Higher Education, Institute of Precision Medicine, Jining Medical University). The NCM460, SW480, HCT8, HCT116, Caco2, and HT29 cells were maintained in RPMI-1640 (Biological industries) with 10% FBS. All cells were cultured at 37°C with 5% CO_2_.

### RNA extraction and quantitative reverse transcription polymerase chain reaction (RT-PCR)

Total RNA was isolated by Trizol reagent (Vazyme) and performed reverse transcription PCR with Go Script reverse transcription system (Promega). On the ABI Prism 700 thermal cycler, real-time qPCR was performed using GoTaq qPCR Master Mix (Promega). The following is the primer sequence: *RAB3IP* (forward primer: AAGCTGAAGTAGCTGCATTGAA; reserved primer GCCACTCATAGCACTGCT TGT); *GAPDH* (forward primer: CTGGGCTAC ACTGAGCACC; reserved primer: AAGTGGTCGTTGAGGGCAATG). GAPDH was used as an internal control.

### Wound-healing assay

Inoculate 2×10^5^ SW480 and HCT8 cells in a 6-well plate, and when they grow to logarithmic phase and close to 90% confluence, draw a straight line vertically in the center of the plate with a pipette tip. After washing three times with PBS, transfect the RAB3IP overexpression plasmid. Choose 0h, 24h, 48h three-time points to take pictures, and finally use the Image J software to calculate the distance of cell migration (Cultivate with the serum-free medium during the experiment).

### Statistical analysis

One-way analysis of variance (ANOVA) was used to analyze the expression levels of colorectal cancer patients in the high-risk group and the low-risk group (TCGA data set), and multivariate analysis of variance was performed to determine the grade, age and survival status of patients with CRC expression levels. ANOVA was used to analyze the characteristic risk scores and clinical or molecular pathological characteristics of colorectal cancer. The ROC curve was used to test the prediction efficiency of the risk signature. The Kaplan-Meier method was used for OS in low-risk and high-risk groups. Chi-Square test was used to conduct statistical analysis on the relationships between the mRNA expression of SFs and the clinicopathological characteristics of patients with CRC. All statistical analyses were performed using Rv3.4.1 (https://www.r-project.org/) and Prism 8 (GraphPad Software Inc.) software.

## Results

### Development of a risk signature by alternative splicing profiles in CRC

After analyzing the raw data used in CRC, we observed ESs in 1637genes, ATs in 1198 genes, APs in 552 genes, RIs in 310 genes, AAs in 220 genes and ADs in 207 genes. Among all AS events, ES is the most common, whereas ME is the least frequent. Notably, a single gene may undergo multiple splicing modes; therefore, we visualized all AS events using an UpSet diagram (Figure S1A).

**Figure 1:**
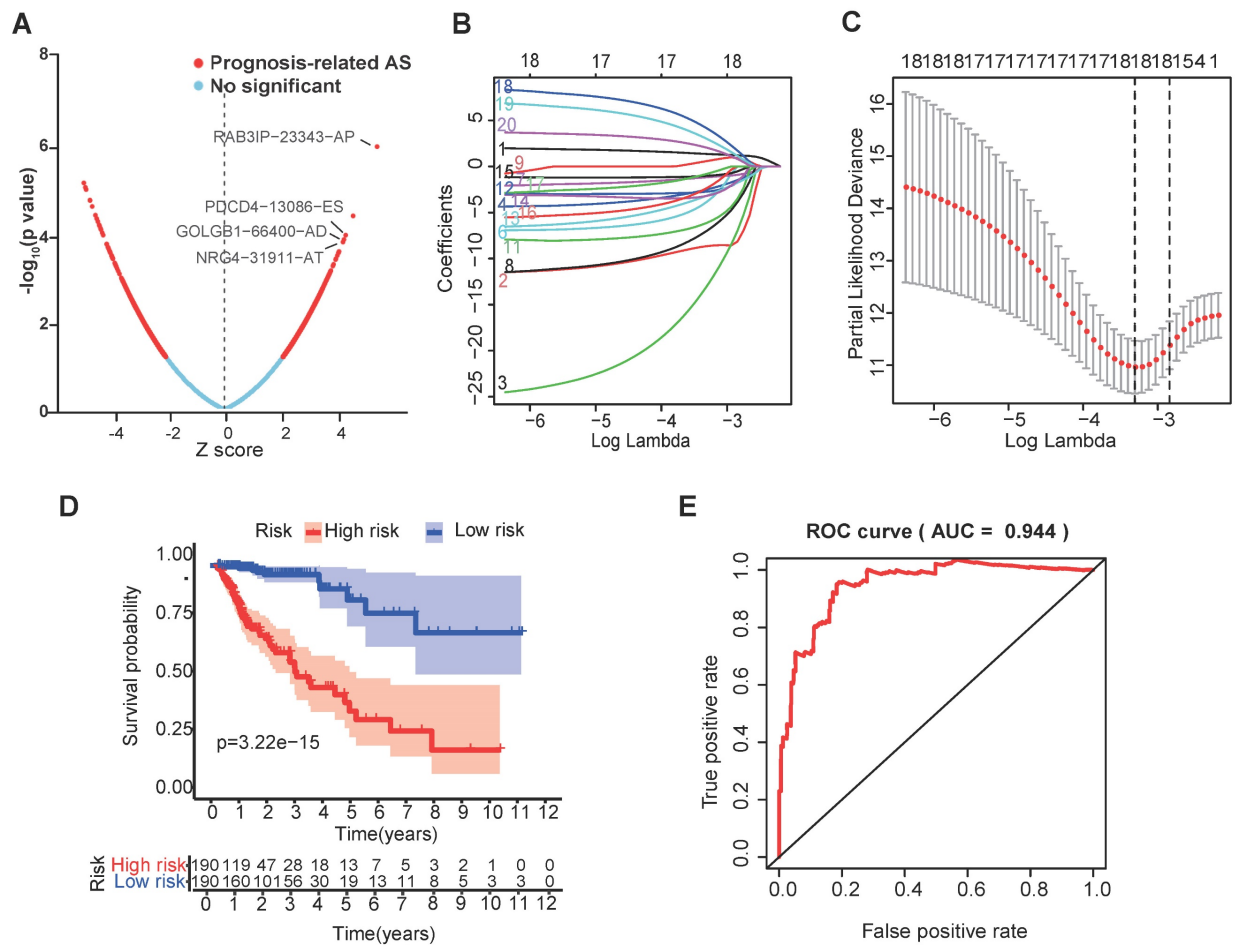
** Risk signature with splicing factor (SF). (A)** LASSO regression analysis of the 2309 SFs. **(B)** Tenfold cross-validation for tuning the parameter selection in the LASSO regression. The solid vertical lines indicate the partial likelihood deviance with standard error. The dotted vertical lines represent the optimal values of the tuning parameter (λ) by minimum criteria. **(C)** Kaplan-Meier OS curves for patients in the TCGA datasets designated to high and low-risk groups depended on the risk score. **(D)** ROC curves demonstrated the predictive efficiency of the risk signature in the CRC of TCGA datasets.

To investigate the association between AS events and CRC survival, we conducted univariate Cox regression analysis and identified survival-related alternative splicing factors (Figure 1A). At the same time, the Upset chart presented AS events related to survival (Figure S1B). In order to better predict the clinical outcome of CRC with alternatively spliced genes, we used the least absolute shrinkage and selection operator (LASSO) Cox regression algorithm for 2309 *SF*s in the TCGA data set, and obtained the risk score through R packages LASSO regression analysis (Figure 1B, C). Using the median risk score (median risk score = 1.09) as the cut-off point, we divided all patients into a high-risk group and a low-risk group, and carefully studied the significant difference in OS between the two groups (P <0.05; Figure 1D). Receiver operating characteristics (ROC) analysis is used to test whether survival predictions are sensitive and specific based on risk scores. The calculation of the area under the curve (AUC) value is based on the ROC curve (Figure 1E). The ROC curve shows that the supporting result of our risk model is AUC = 0.944.

### Verification of a risk signature consisting of four SFs

**Figure 2:**
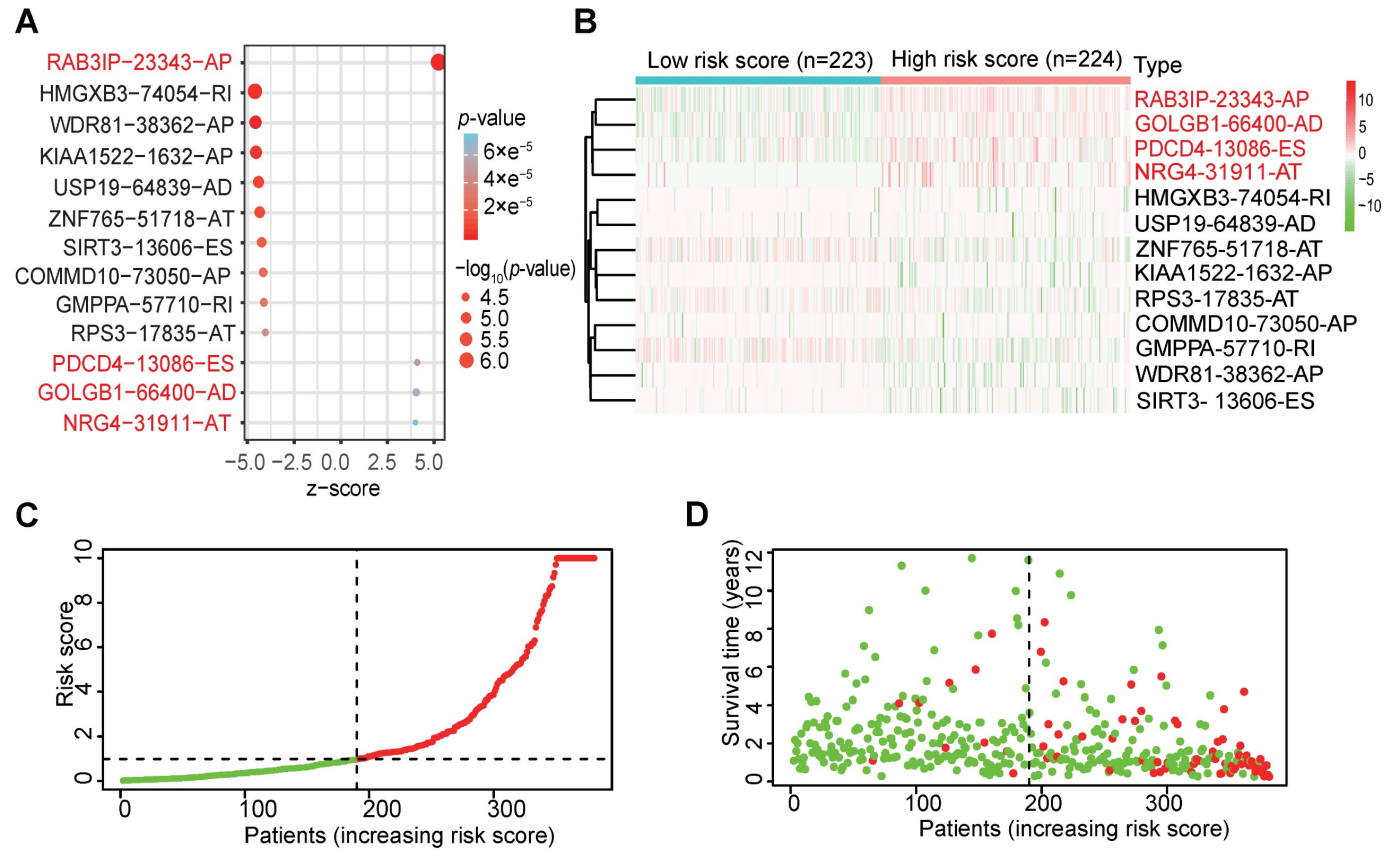
** The selection of four SFs and their effect on CRC prognosis. (A)** Cox univariate regression analyses were used to examine the associations between expression of 13 SFs and prognosis. The P-value of RAB3IP, PDCD4, GOLGB1 and NRG4 < 0.05. Z-score: (z-score standardization), represent the correlation with the P-value. **(B)** Heatmap of the 13 selected SFs. **(C-D)** Risk score and survival status for each patient.

We further investigated the prognostic value of a single alternative splicing factor in CRC. We performed univariate Cox regression analysis on the expression level of each alternative splicing factor in different alternative splicing events in the TCGA data set. The results showed that among the 13 alternative splicing factors with the smallest P-value, 4 SFs were significantly related to OS (Table 1) (P<0.05; Figure 2A; Figure S2), and these four alternative splicing factors were highly expressed in high-risk patients (Figure 2B). Then, we analyzed the distribution of risk scores and survival status (Figure 2C, D). In Figure 2C, the risk score of each patient is ranked from low to high, and the patients are divided into low-risk groups (green dots) and high-risk groups (red dots) according to the median risk score. High-risk patients demonstrated significantly increased mortality (58.3% vs. 22.7%, P<0.001) with earlier median survival time (32 vs. 68 months, log-rank P<0.001) compared to low-risk counterparts (Figure 2D).

### Independent prognostic analysis

**Figure 3:**
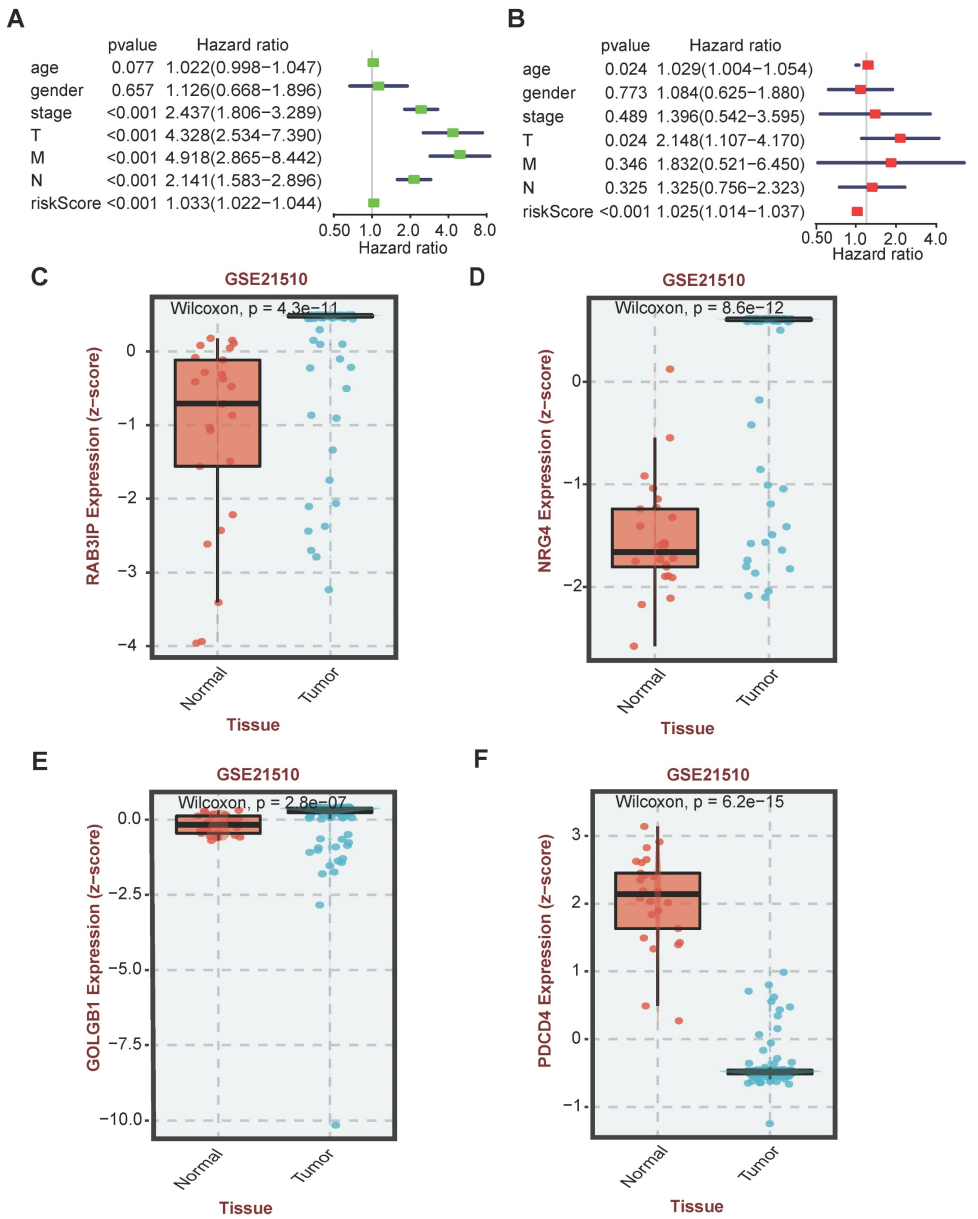
** Independent analysis of clinicopathological characteristics of four SFs. (A)** Univariate analysis of the hazard ratios for risk score as independent prognostic elements to anticipate the OS. **(B)** Multivariate analysis of the hazard ratios for risk score as independent prognostic elements to predict the OS.** (C-F)** Expression profile analysis of four SFs in the GEO database.

**Figure 4:**
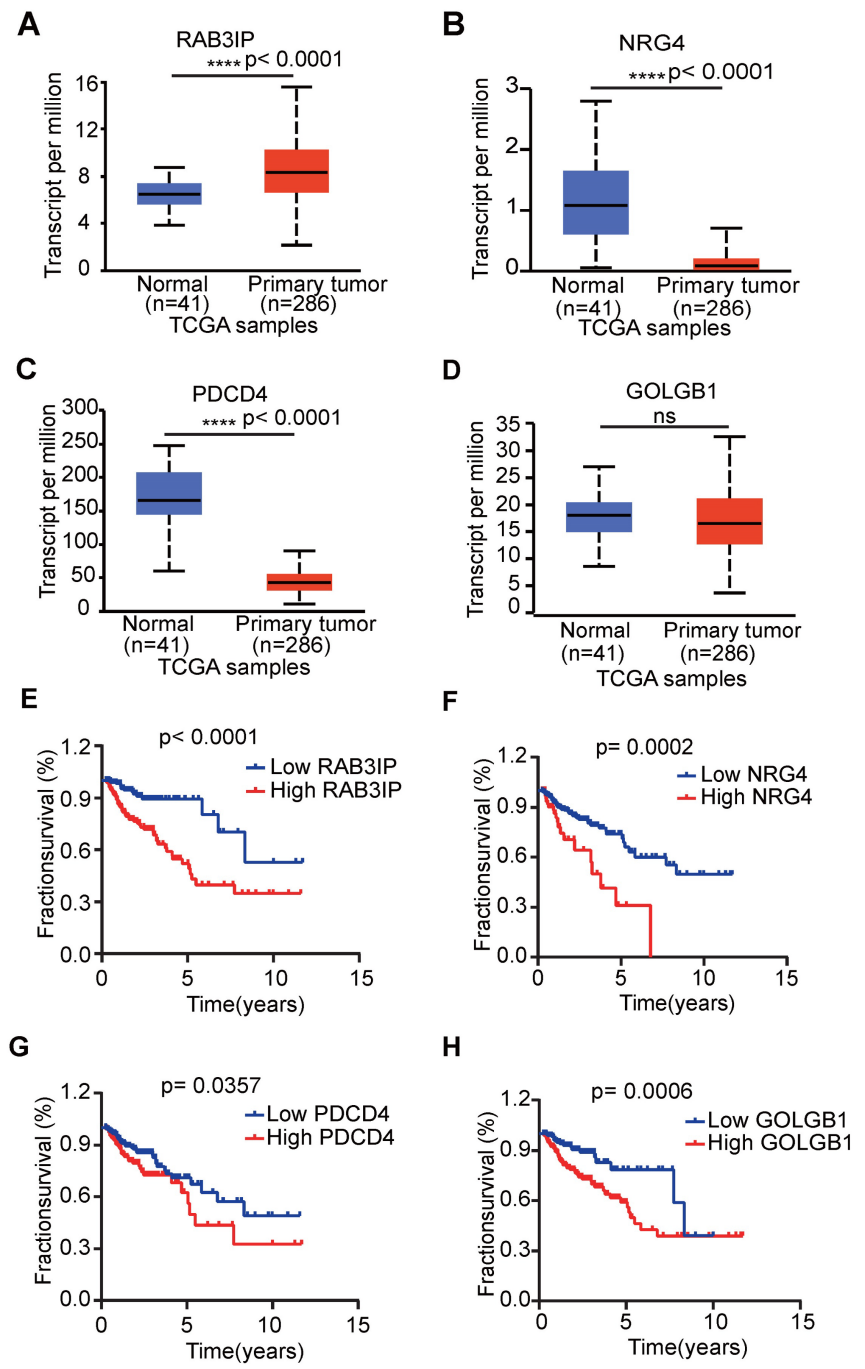
** Expression profile and OS analysis verify the effect of four SFs on tumor progression. (A-D)** Expression profile analysis of four SFs in the TCGA database; **(E-H)** OS survival curves of CRC patients based on four selected SFs.

In order to verify whether the clinicopathological characteristics (including age, gender, stage, depth of tumor invasion, local lymph node enlargement, and distant metastasis) are independent prognostic factors for the patient's prognosis, we conducted a univariate and multivariate analysis of OS. Univariate analysis using the Cox proportional hazards model for all variables showed that risk score (P <0.001, 95% CI HR 1.022-1.044) can be used as an independent factor for poor prognosis of CRC patients (Figure 3A). The same multivariate analysis as the cohort univariate analysis supports that risk score (P = 0.001, 95%CI HR 1.014-1.037) and age (P = 0.024, 95%CI HR 1.004-1.054) are independent factors for the poor prognosis of CRC patients, but there is no obvious correlation between TNM stage, tumor invasion depth, regional lymph node metastasis and distant metastasis. To further identified the correlation between the alternative splicing factors of RAB3IP, NRG4, PDCD4 and GOLGB1 and tumor progression, the expression of four SFs in CRC tissue was analyzed using BEST. Consistent with our earlier result from the TCGA dataset, the expression of RAB3IP, NRG4 and GOLGB1 showed higher level in CRC tissue in GEO (Figure 3C-E). Surprisingly, a negative correlation was observed between the mRNA expression levels of PDCD4 and tumor progression as shown in Figure 3F. In addition, we analyzed the expression profiles of RAB3IP, PDCD4, GOLGB1 and NRG4 in Normal and Primary tumors in the TCGA database on the UALCAN website(http://ualcan.path.uab.edu), The results showed that RAB3IP was highly expressed in tumors, and NRG4 and PDCD were downregulated in tumors. There was no significant difference in GOLGB1 (Figure 4A-D). At the same time, we compared the OS of patients with high expression levels of these four genes with those with low expression levels and found that the high expression of RAB3IP, NRG4, PDCD4 and GOLGB1 had poor prognostic effects (Figure 4E-H).

Furthermore, we expanded our analysis by including 447 CRC patient samples from the TCGA database to investigate the correlations between the four SF genes and the pathological characteristics of CRC (Table 2). The results revealed significant associations between the expression of RAB3IP and NRG4 and various clinicopathological parameters in CRC patients. We observed that high levels of RAB3IP expression were significantly associated with the Lymph node metastasis of CRC patients. The expression of NRG4, PDCD4 and GOLGB1 had no obvious relevance of CRC. Additionally, high expression levels of RAB3IP were significantly associated Overall survival. These findings suggest that RAB3IP may serve as a potential biomarker for CRC, indicating its diagnostic relevance. Based on the concordant evidence of tumor-specific overexpression and strong survival association, RAB3IP was prioritized for functional characterization (Figure 4A, E).

### The function of RAB3IP in regulating migration of CRC cells

**Figure 5:**
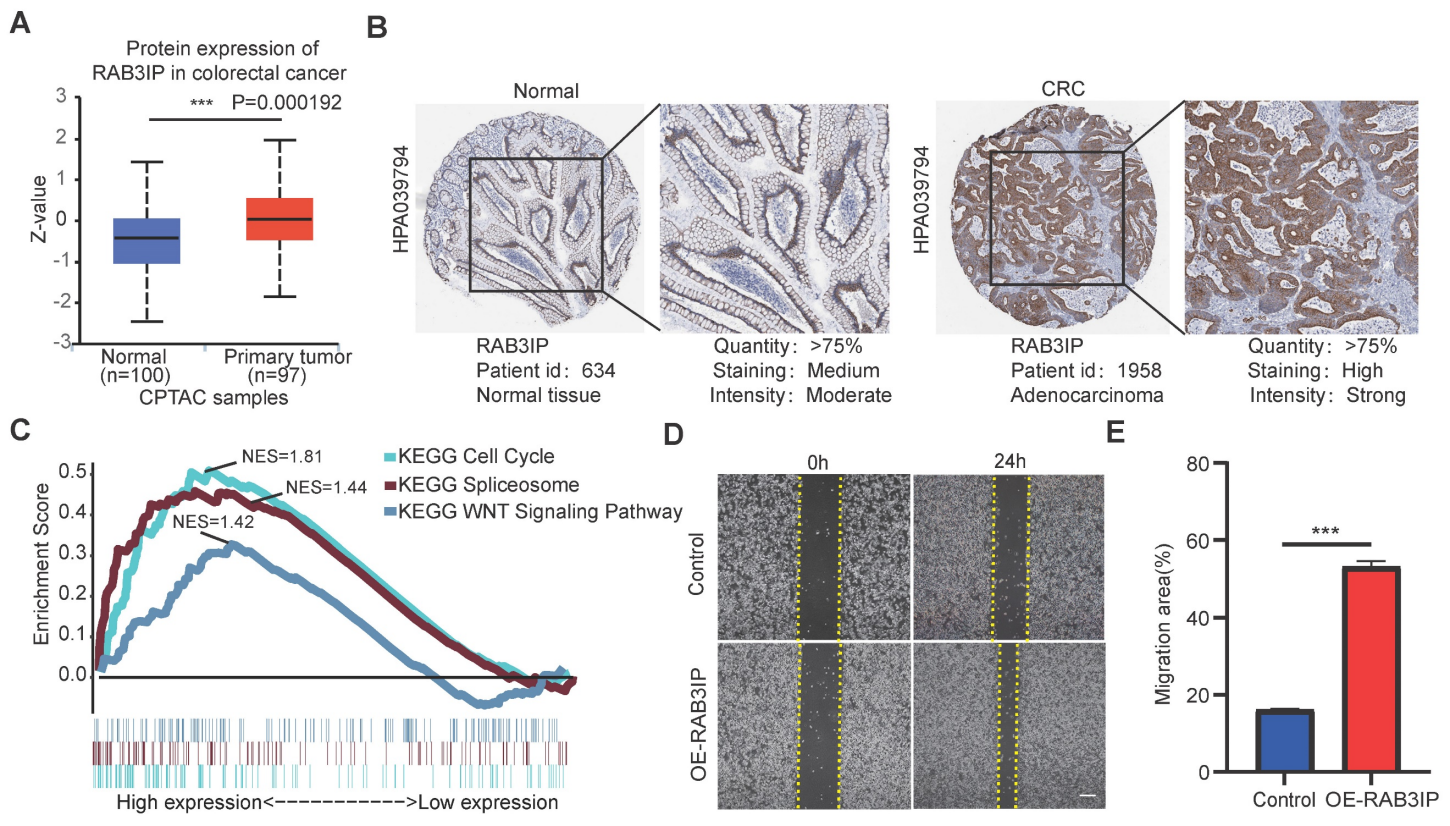
** RAB3IP function verification and pathway enrichment. (A)** Based on the CPTAC database, analyze the expression of RAB3IP protein in Normal and Primary tumors; **(B)** Use the Human Protein Atlas database to validate RAB3IP through immune tissue chip;** (C)** Enrichment of genes in the Kyoto Encyclopedia of Genes and Genomes (KEGG) different pathways by GSEA; **(D)** Overexpression of RAB3IP promoted SW480 migration as analyzed by scratch wound assay. Scale bar: 200μm. (B) Quantitative analysis of the migration rates in (A). n = 3 per group.

In order to clarify the important function of RAB3IP in CRC, we downloaded its protein expression in normal and primary tumors from the CPTAC database, and the results are consistent with the mRNA expression profile (Figure 5A). Additionally, tissue microarray data from the Human Protein Atlas database indicated that RAB3IP is upregulated in tumor tissues, suggesting its potential role in tumor progression (Figure 5B). Next, we used gene enrichment analysis (GSEA) and found that RAB3IP is related to Cell Cycle, WNT pathway and Spliceosome (Figure 5C). To further clarify the role of RAB3IP in colorectal cancer, first, we examined the expression of RAB3IP in a variety of colorectal cancer cells. Among them, SW480 and HCT8 show relatively low levels of *RAB3IP* transcripts (Figure S3A). We studied the biology of *RAB3IP* in CRC pathology by overexpressing RAB3IP cDNA in those two cell lines and conducted Wound-Healing Assays. Our results indicated that after overexpression of RAB3IP, the migration speed of SW480 was increased compared with the control by 27% (Figure 5D, E). The enhancement trend was remained in HCT8 cell (Figure S3B).

## Discussion

Aberrant mRNA splicing is recognized as a critical driver of carcinogenesis and tumor progression 25, 26. Although AS events have been characterized in numerous genes, their functional significance remains poorly understood in the global landscape of alternative splicing. With the advancement of high-throughput sequencing technologies and bioinformatics data analysis methods, we have more resources to analyze the effects of different AS events on the occurrence and development of cancer.

Colorectal cancer (CRC), ranking as the fourth leading cause of cancer-related mortality worldwide, demonstrates strong associations with dysregulated alternative splicing 17, 18. Therefore, it is particularly important to analyze the AS events in CRC deeply and clarify the macroscopic prognostic criteria of splicing factors in AS events in the development of tumors 27. In this study, we determined the AS events in colorectal cancer transcriptome through the overall analysis of RNA-seq data and clinical data in the TCGA database and found that ESs in 1637 genes, ATs in 1198 genes, APs in 552genes, RIs in 310, AAs in 220 genes and ADs in 207 genes. Additionally, there are many genes that have more than one alternative splicing variant. Then, we first selected four (RAB3IP, PDCD4, GOLGB1 and NRG4) and prognostic-related alternative splicing factors through Cox univariate analysis and LASSO Cox regression analysis, and conducted risk and post-analysis. Next, we used univariate analysis and multivariate analysis to evaluate the prognostic value of these four splicing factors and found that all were positively correlated with poor prognosis, aligning with multiple previous research outputs 28-34. It is worth noting that this is not consistent with the fact that most of the survival-related AS events in ovarian cancer are favorable prognostic factors 35. In addition, there is reported that PDCD4 is transcriptionally repressed by the alternative splicing factor SRSF3, which promotes the metastasis of colorectal cancer 36, 37. Breast cancer exosomes contribute to pre-metastatic niche formation and promote bone metastasis of tumor cells via the miR21-PDCD4 axis 33. Meanwhile, it has also been found that EMT-Exos-derived exosomal miR-106b can inhibit the expression of PDCD4 in macrophages, induce M2 macrophage polarization by activating PI3Kg/AKT/mTOR pathway, and promote the malignant progression of CRC cells 38. NRG4 has been identified and characterized as a novel variable shear factor in prostate cancer, but its specific role remains unclear 29. In 2014, GOLGB1 was reported to enhance susceptibility to galectin-1 in prostate cancer, thereby inducing cellular apoptosis 31. Recent research also demonstrates that GOLGB1 plays a critical role in the risk of pulmonary metastasis among breast cancer patients 28. For RAB3IP, there are much evidence demonstrate that it can promote cell proliferation and metastasis in many cancers, but its role as an alternative splicing factor in CRC remains unclear 30, 32.

**Figure 6:**
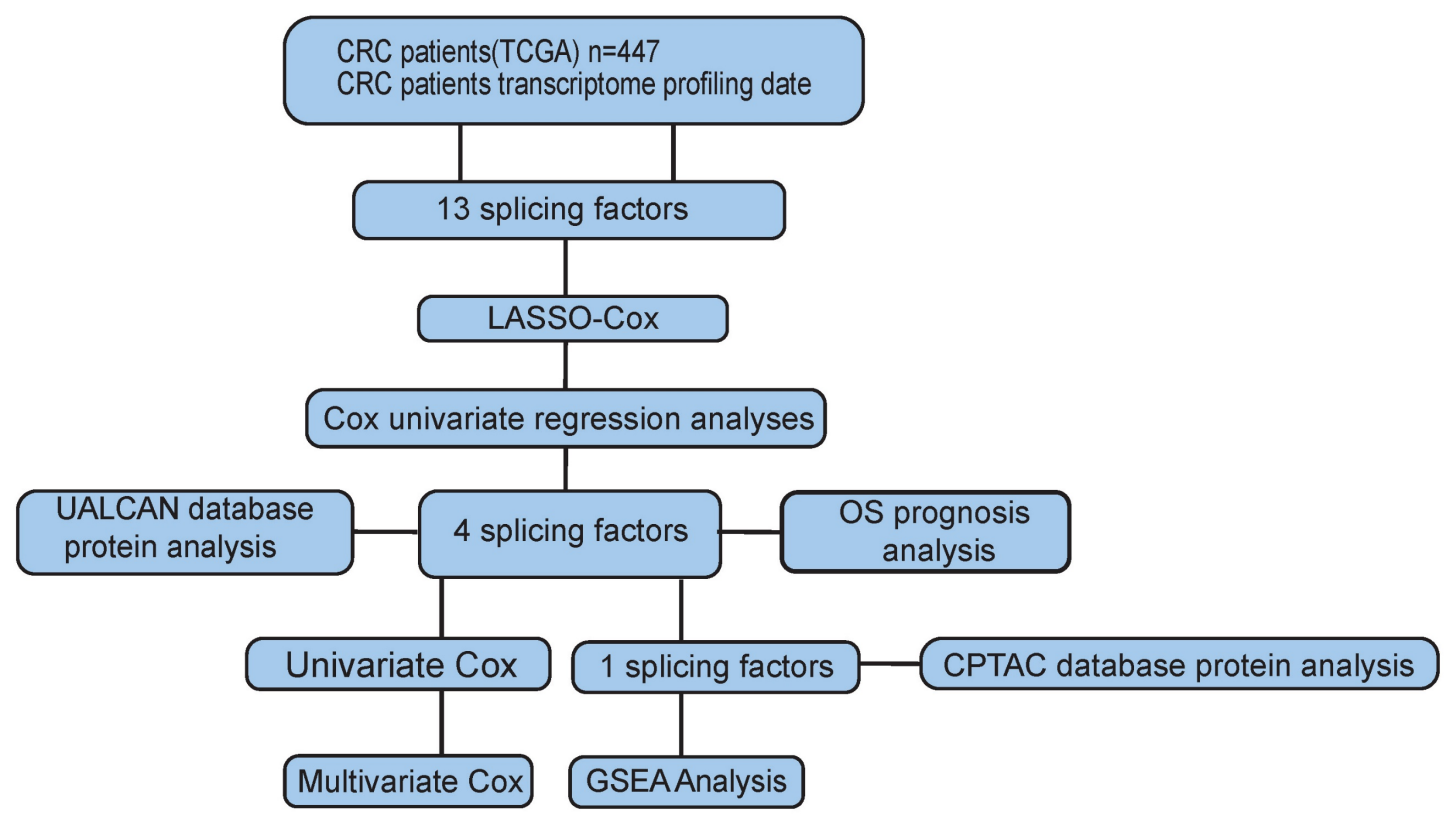
Workflow chart of data generation and analysis.

Then, we analyzed the mRNA and protein expression of the four genes in normal tissues and tumors and found that only RAB3IP was significantly upregulated in tumors, further supporting its correlation with tumor occurrence and development in CRC. Finally, through gene chip analysis and GSEA analysis, it revealed the enrichment of RAB3IP in Cell Cycle, WNT, and Spliceosome pathways. All the technical routes are shown in Figure 6. Other studies have shown that RAB3IP is highly expressed in gastric cancer and induces the migration and invasion of gastric cancer cells through the EMT pathway 39, 40. But whether it has the same effect on CRC remains to be further studied.

In conclusion, this study not only provides a comprehensive characterization of AS factor expression and prognostic value in CRC but also establishes a framework for AS-related cancer research. It also provides new biomarkers for predicting the prognosis of CRC, and may even provide new targets for treatment development.

## Conclusion

In conclusion, our study highlights the clinical significance and prognostic value of AS events in CRC. Moreover, we further identified RAB3IP as an alternative splicing factor affecting CRC progression by affecting Cell Cycle, WNT, and Spliceosome pathways. These findings not only help to construct an association between AS events and CRC metastasis, but also provide novel targets for future anti-metastasis therapies.

## Supplementary Material

Supplementary figures.

## Figures and Tables

**Table 1:** List of 13 most differential alternative splicing (AS) factors in CRC

ID	HR	HR.95 L	HR.95 H	pvalue
**RAB3IP-23343-AP**	61.89	12.71	301.41	3.26×e^-7^
HMGXB3-74054-RI	1.25×e^-8^	6.38×e^-12^	2.46×e^-5^	2.57×e^-6^
WDR81-38362-AP	1.73×e^-8^	9.24×e^-12^	3.23×e^-5^	3.31×e^-6^
KIAA1522-1632-AP	0.0074	0.00093	0.059	3.73×e^-6^
USP19-64839-AD	3.58×e^-6^	1.51×e^-8^	0.00085	6.97×e^-6^
ZNF765-51718-AT	0.0093	0.0012	0.074	9.78×e^-6^
SIRT3-13606-ES	8.68×e^-7^	1.53×e^-9^	0.00049	1.60×e^-5^
COMMD10-73050-AP	5.12×e^-9^	7.45×e^-13^	3.52×e^-5^	2.28×e^-5^
GMPPA-57710-RI	0.0072	0.00072	0.073	2.92×e^-5^
RPS3-17835-AT	8.25×e^-6^	3.01×e^-8^	0.0023	4.36×e^-5^
**PDCD4-13086-ES**	61502.12	298.28	12681030.40	5.00×e^-5^
**GOLGB1-66400-AD**	575.23	25.52	12963.56	6.38×e^-5^
**NRG4-31911-AT**	39.70	6.43	245.01	7.36×e^-5^

Bold font indicates significant HR; HR, hazard ratio;

**Table 2:** Relationship between clinicopathologic parameters and four SF expression in CRC

Variable	RAB3IP expression		*P* value	NRG4 expression		*P* value	PDCD4 expression		*P* value	GOLGB1 expression		*P* value
	Low (n=239)	High (n=208)		Low (n=323)	High (n=124)		Low (n=248)	High (n=199)		Low (n=236)	High (n=211)	
Sex, N (%)			0.9468			0.6729			0.7925			0.8389
M	126(52.72)	109 (52.40)		172 (53.25)	63 (50.81)		129(52.02)	106 (53.27)		123(52.12)	112 (53.08)	
F	113(47.28)	99 (47.60)		151 (46.75)	61 (49.19)		119(47.98)	93 (46.73)		113(47.88)	99 (46.92)	
Age at diagnosis, N (%)			0.9308			0.244			0.5502			0.6552
<50	28(11.72)	24(11.54)		33 (10.22)	18(14.52)		26(10.48)	25(12.56)		25(10.59)	26(12.32)	
≥50	211(88.28)	184(88.46)		290 89.78)	106(85.48)		222(89.52)	174(87.44)		211(89.41)	185(87.68)	
TNM Stage, N (%)			0.1609			0.4189			0.5809			0.7014
I-II	132(55.46)	129(62.02)		66(20.31)	21(16.94)		50(20.16)	36(18.09)		47(19.92)	39(18.48)	
III-IV	106(44.54)	79(37.98)		259(79.69)	103(83.06)		198(79.84)	163(81.91)		189(80.08)	172(81.52)	
Lymph node metastasis, N (%)			**0.0091**			0.4959			0.9568			0.3029
Absent	113(47.28)	87(35.10)		176(54.49)	72(58.06)		112(44.98)	89(44.72)		111(47.03)	89(42.18)	
Present	126(52.72)	121(64.90)		147(45.51)	52(41.94)		137(55.02)	110(55.28)		125(52.97)	122(57.82)	
Overall survival, N (%)			**0.0104**			**0.0288**			0.3679			0.2441
Alive	209(87.45)	163(79.33)		284(87.93)	99(79.84)		219(88.31)	170(85.43)		211(89.41)	181(85.78)	
Dead	30(12.55)	45(20.67)		39(12.07)	25(20.16)		29(11.69)	29(14.57)		25(10.59)	30(14.22)	

Bold font indicates significant difference.

## Abbreviations

ROC: Receiver Operating Characteristic
AUC: Area under curve
GSEA: Gene Set Enrichment Analysis
UALCAN: The **U**niversity of Alabama at Birmingham Cancer data analysis Portal
CPTAC: The National Cancer Institute's Clinical Proteomic Tumor Analysis Consortium
KEGG: Kyoto Encyclopedia of Genes and Genomes
