# Supplementary material for: Alternative splicing factor RAB3IP as a novel risk signature to predict the prognosis of colorectal cancer
Source: J Cancer. 2025 Jun 23;16(9):2959–69. doi: 10.7150/jca.110271 (PMC12244333; doi:10.7150/jca.110271)
Supplement: Supplementary file 1 — Supplementary figures. [file jcav16p2959s1.zip › Supplementary Materials.docx]

**
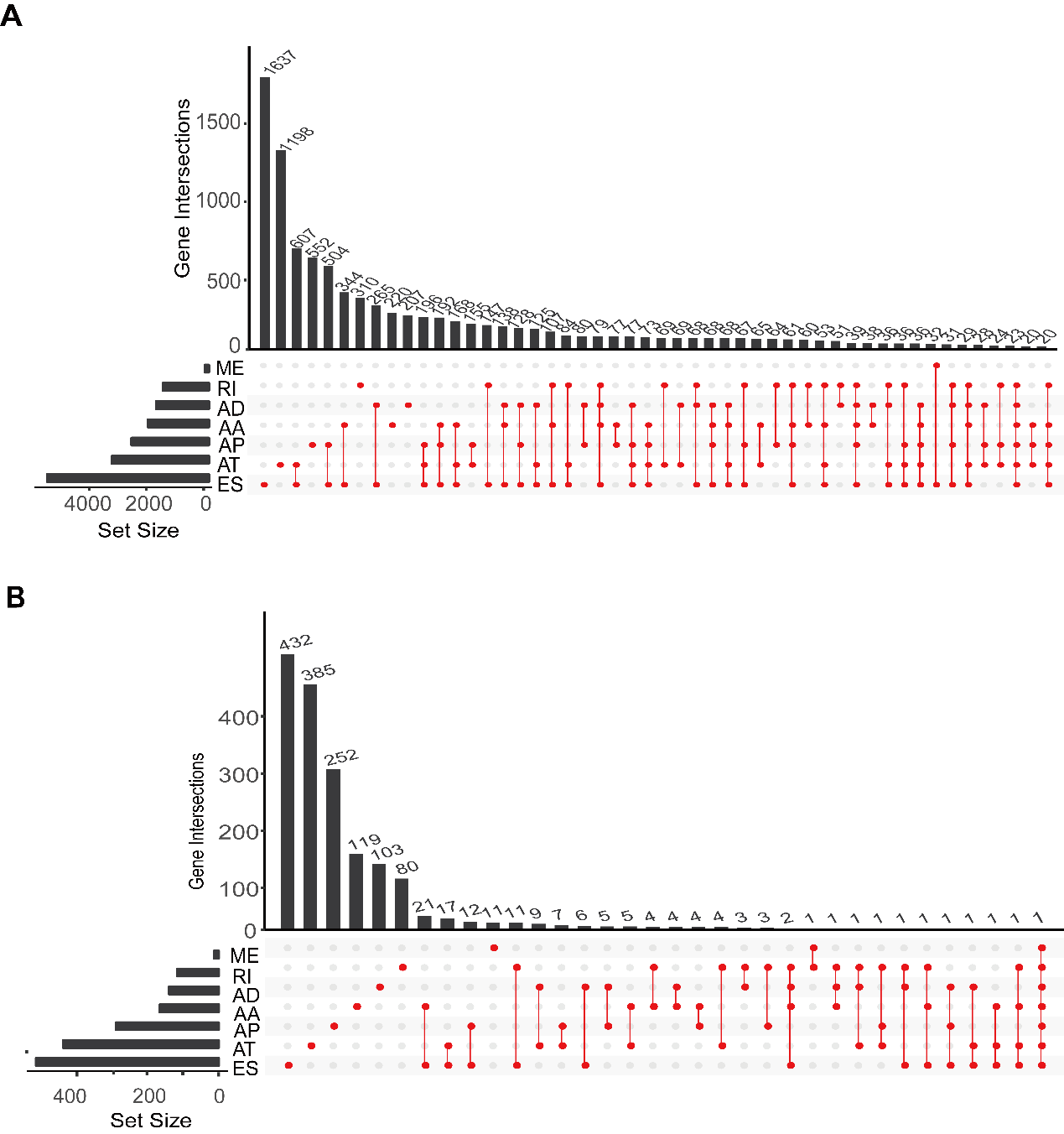
**

**Supplementary Figure 1 Upset plot of different types of alternative splicing types.** (A) The UpSet plot shows seven types of AS events in CRC. (B) The UpSet plot shows seven types of survival-associated AS events in CRC.

**
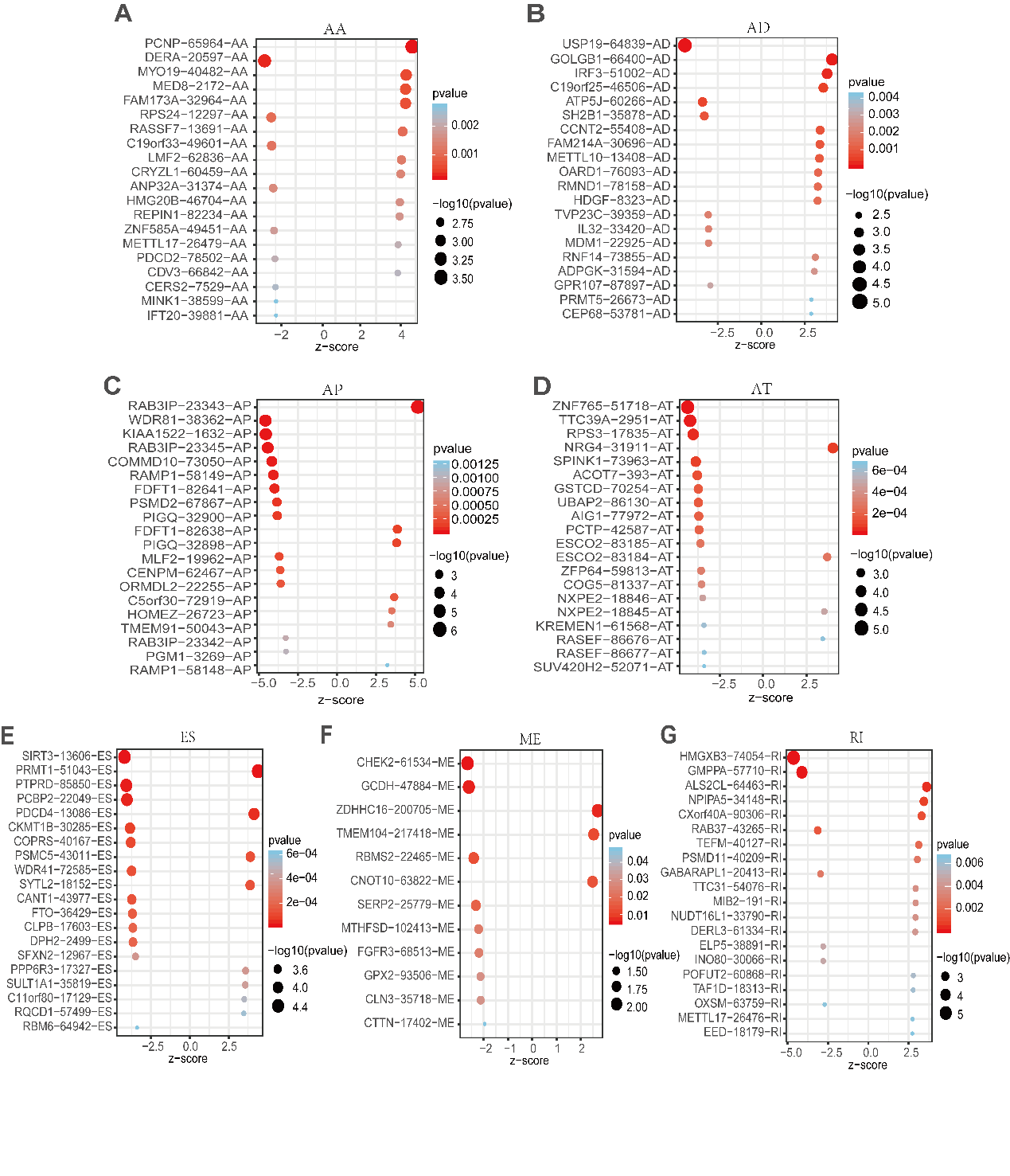
**

**Supplementary Figure 2 Bubble chart for subgroup analyses of survival associated AS events in CRC cohort.** (A–G) Bubble chart of HRs for survival associated AA, AD, AP, AT, ES, ME and RI events in CRC, respectively. The shade color scale of the bubble color represents the P-value. The size of the circles represents -log10(p-values) by the side.

**
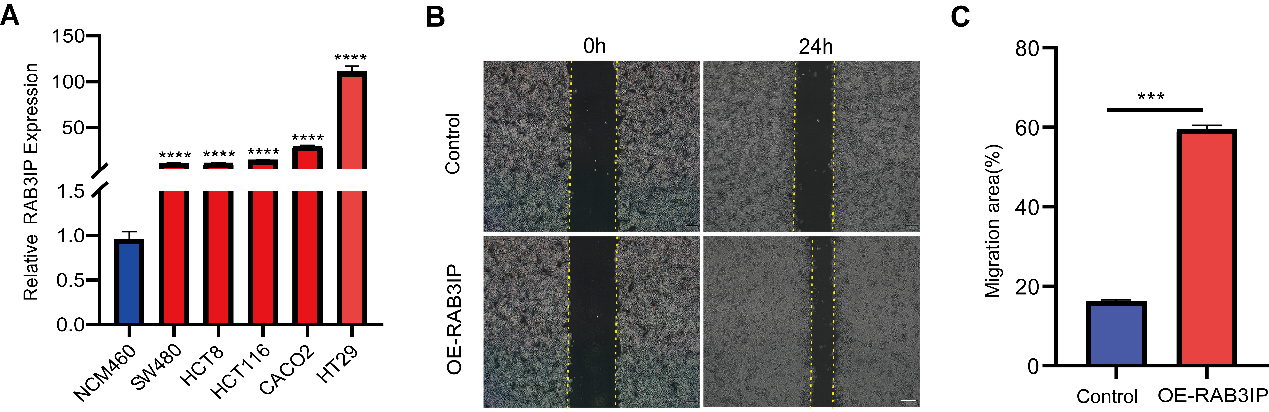
**

**Supplementary Figure 3 Relative expression and functional verification of RAB3IP in different cell lines** (A) Relative expression of RAB3IP in different cell lines; (B) Overexpression of RAB3IP promoted HCT8 migration as analyzed by scratch wound assay. Scale bar: 200 μm. (C) Quantitative analysis of the migration rates in (A). n = 3 per group.
