# Supplementary figures and images for: Alternative splicing factor RAB3IP as a novel risk signature to predict the prognosis of colorectal cancer
Source: J Cancer. 2025 Jun 23;16(9):2959–69. doi: 10.7150/jca.110271 (PMC12244333; doi:10.7150/jca.110271)

**A**

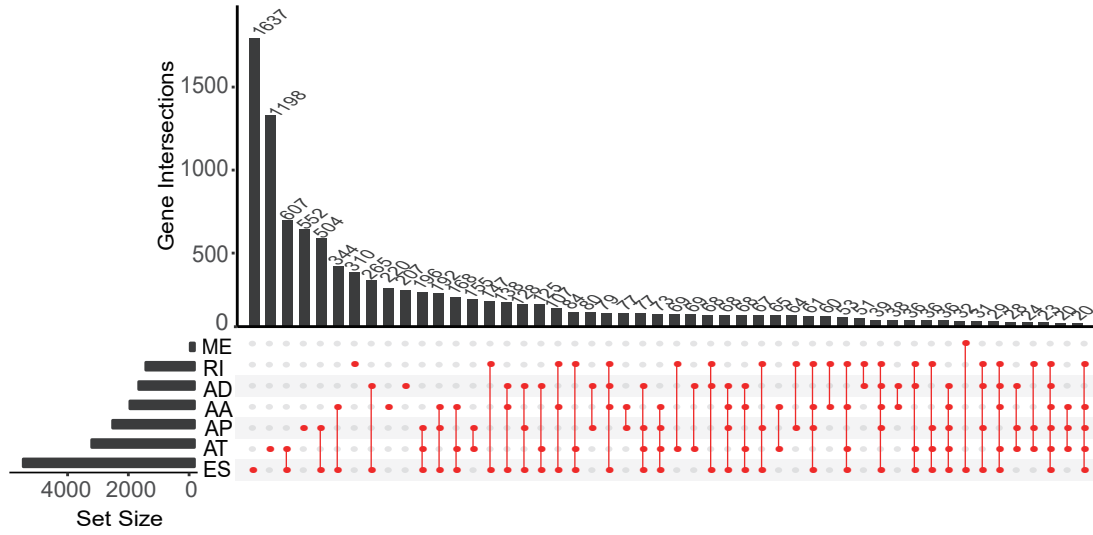

**B**

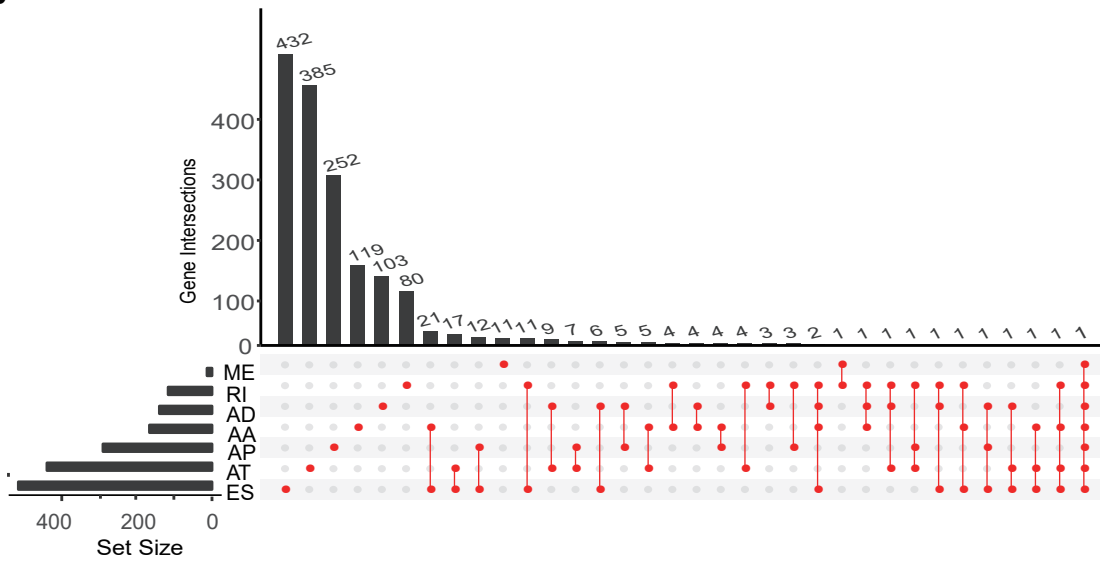

A

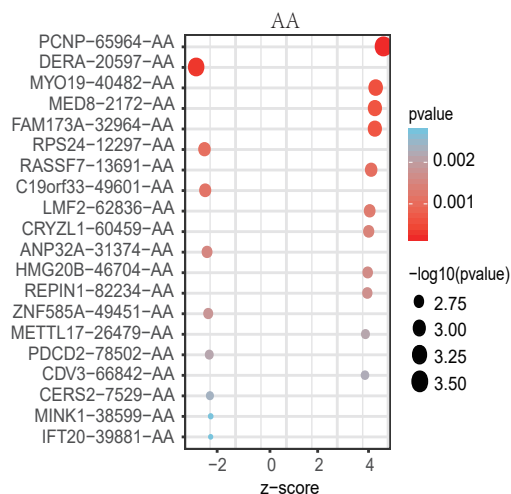

B

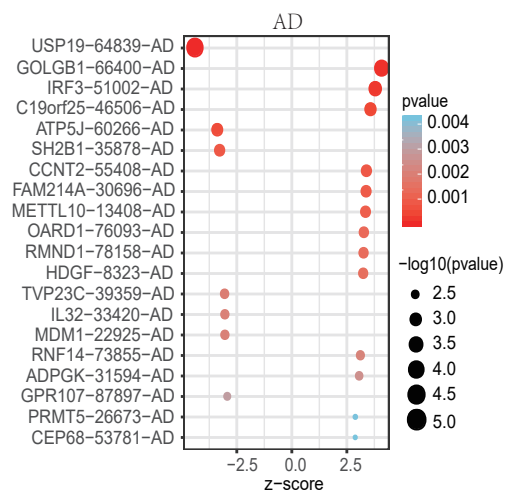

C

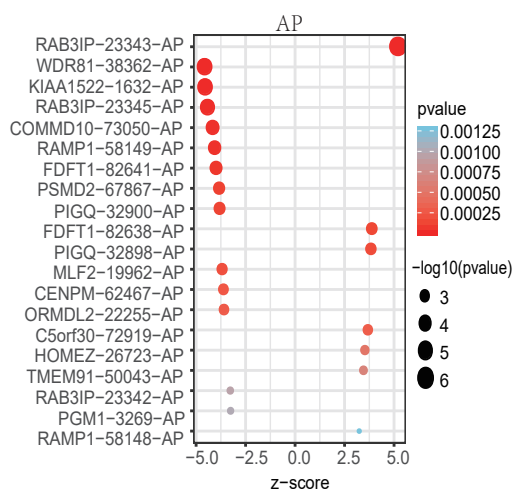

D

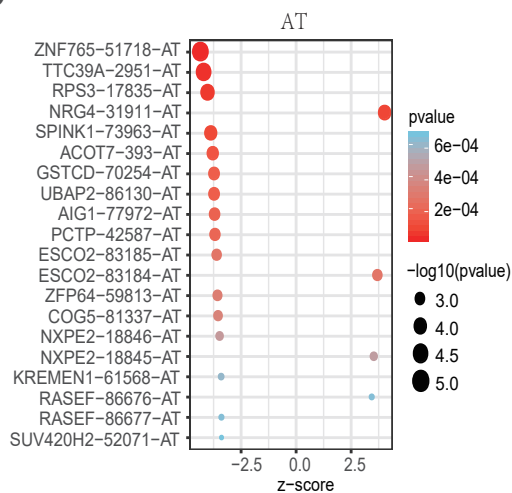

E

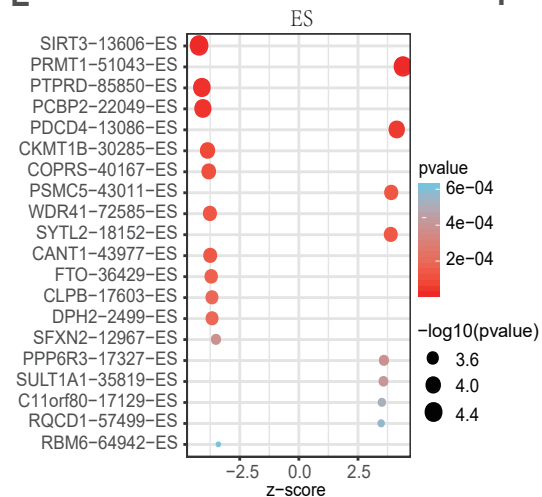

F

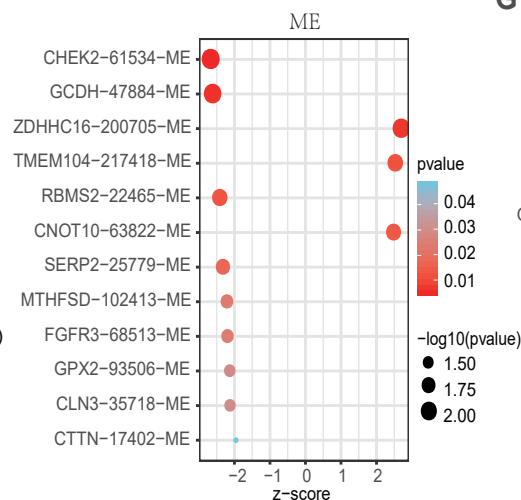

G

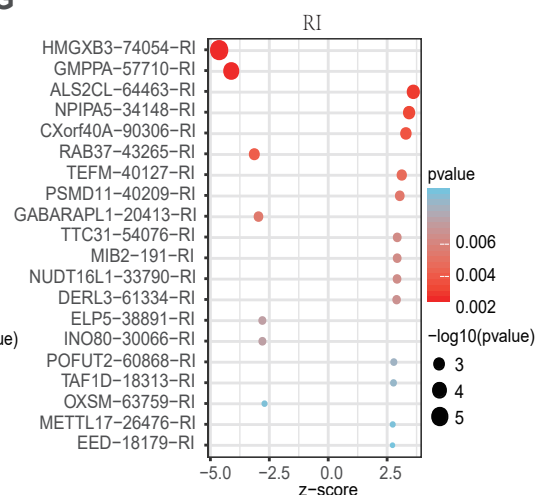

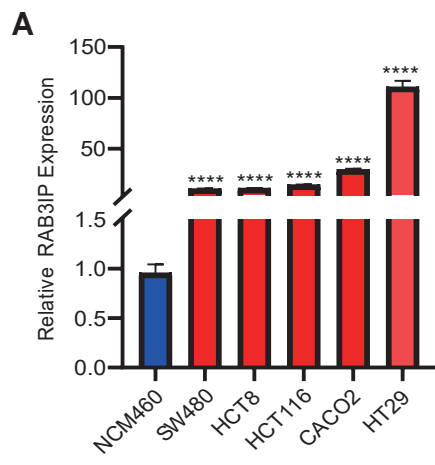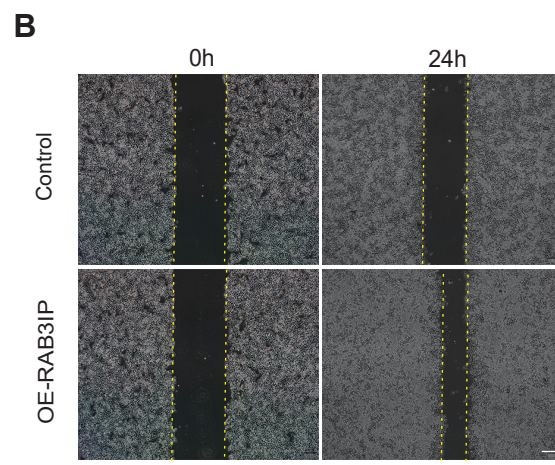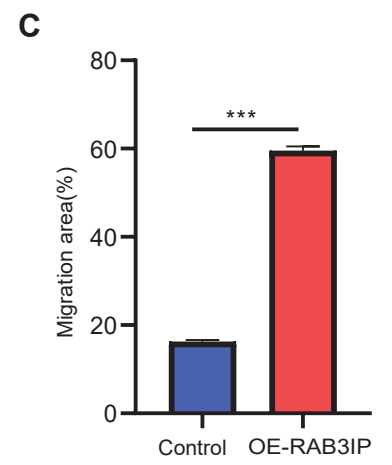

Supplement: Supplementary file 1 — Supplementary figures. [file jcav16p2959s1.zip › supplement figure .pdf]
